# The Ordeal of Burial

**Published:** 1892-01-23

**Authors:** 


### PASSING TOPICS.

### THE ORDEAL OF BURIAL.

The question of the propriety of insisting that a large portion of the burial service shall be read at the grave side is again being raised, and not too soon or too vehemently. It is high time people spoke out, and in no uncertain tone. If it be possible that the remembrance of the painful calamity which has just plunged the nation in sorrow can be made to serve any good purpose, it is assuredly by keeping sight of the fact that there is reason for believing the life of the Duke of Clarence was sacrificed to the preposterous requirement that mourners shall stand bareheaded around the open grave of some lost one committed to the earth amid the inclemencies of our English weather.

It is not as if east wind, rain, sleet, and other dangers and discomforts were abnormal contingencies to be safely overlooked in a majority of instances. We all know that there are but few days in the course of a year which can be accounted safe for any open-air functions, except of an active and rousing kind. Young, middle-aged, and elderly people who are careful of warmth and covering at all other times, find themselves compelled either to forego participation in the last tribute of respect or affection to a friend or relation or to place in jeopardy their own lives. This is not to put the case too strongly. Large numbers of persons cannot doff their headgear in the keen air even for the space of a few moments without suffering, and ten minutes or more of uncovering, such as the burial ceremonial now demands, constitutes a sufficient space of time wherein to contract the most serious consequences, and not by weakly persons alone. Added to this, our cemeteries, ill-drained as most of them are, remain swampy at nearly all seasons, and the neighbourhood of a newly-opened grave, which is seldom accessible except by passing over wet grass, is oftener than not a veritable quagmire. Few more dangerous ordeals than attendance at a funeral in one of our suburban burying-grounds can be conceived. None of the elements of risk are wanting, and naturally the chances of harm arising are greatly heightened by those depressing influences which lower the vitality and render the body an easy victim to disease.

The wonder is the clergy do not suffer more severely than they do; but beginning when young it is possible that they become hardened to the exposure. Happily, with most people, attendance at a funeral is not an everyday occurrence, and they have no opportunity, even if they had the will, to take to it with the safety born of habit. If the officiating clergy have no power to alter the prevailing practice, it is high time it was altered for them. The authorities of the Church must not show themselves indifferent to the infirmities of our common humanity, and a very acceptable indication of their solicitude would be such modification of existing regulations as should permit the whole of the beautiful service with which we commit our dead to the tomb to be rendered under cover, and without the distraction caused to the survivors by avoidable physical discomfort and danger. Agricola.

